# On the Social Evils, with a Plan for Their Diminution and a Plea for the Innocent and Helpless

**Published:** 1867-12

**Authors:** George J. Zeigler

**Affiliations:** Philadelphia


ARTICLE LII.

### ON THE SOCIAL EVILS, WITH A PLAN FOR THEIR DIMINUTION, AND A PLEA FOR THE INNOCENT AND HELPLESS.

By GEO. J. ZEIGLER, M.D.

The subject of the limitation of prostitution with its dreadful concomitants, so ably treated of by Prof. ANDREWS, in the last number of your excellent journal, is one that appeals to every true philanthropist, and especially to the members of the medical profession, who are, unfortunately, better practically acquainted with its terrible evils than any other class of men. But, while it is desirable to ameliorate the disorders of the body politic, induced by the illicit intercourse of the sexes, it is much more essential to remove the primary causes of such abnormities, in order to prevent their occurrence and preserve the health and purity of society. This, however, can only be effected by acting in accordance with the natural laws for the development of life and the government of humanity.

Among the many millions of human and other living beings existing in the world there is a certain degree of sexual activity for procreative and other purposes. This sexual instinct is so strong and energetic in the mass of mankind that it cannot be restrained, and in one way or another must be satisfied; for it is a well-known fact that, if these natural desires are not legitimately gratified, irregular indulgences and disorders of various kinds ensue, prominent among which are derangement of the brain, nervous system, genital apparatus, etc., with demoralization, unnatural habits, illicit intercourse, diseases of the sexual organs and general system, and premature destruction of life. Now, as these natural appetites cannot be repressed, they must be regulated, by so reorganizing society as to permit or compel a more regular flow of the sexual current and equable reunion of the sexes.

Prostitution, with its attendant evils, is the more immediate result of a defective state of society, for, though dependent, primarily of course, upon the undue activity of the sexual appetite, it is always increased by the difficulty of legitimate association and the false ideas respecting marriage, in which woman is placed at a great disadvantage. The prevalence of prostitution cannot be wholly attributed to the inherent depravity of those who sink into it, but mainly to necessity and the extraneous force of circumstances, as it is most likely that woman is generally influenced in her sexual relations more by the strength of her sympathies and the warmth of her affections, than the intensity of her animal desires; for, were it otherwise, the world would be sunk in the grossest licentiousness.

Now, as there are comparatively few women who voluntarily become prostitutes, some decided effort should be made to prevent this wholesale sacrifice of so many of the fairest and purest of our kind, for it is a lamentable fact that the majority of those who thus lapse from virtue are the healthiest and handsomest of their sex, strength and beauty being powerful incentives to sensuality and essential elements for the successful pursuit of a disreputable life. Besides, the necessity for some positive effort in this direction is the more imperative, as the evil is not limited to any one class, but involves, to a greater or less extent, the whole human family, society being thus doubly outraged by the withdrawal of so much that is desirable for the legitimate relations of life, and the reactive injury to the body politic from the poison engendered therein by the vicious action of many of its most vigorous members of both sexes. But, for the requisite efficiency, such action must be based upon the fundamental principles of equity and justice, by fully recognizing the correlative rights, duties, and obligations of the respective sexes. That these are entirely too much disregarded at present, is apparent; for, as it now is, a man may corrupt and seduce a woman, debauch and disease her body, impregnate and beget her with child, and then discard her as a strumpet, he being comparatively free to leave her and repeat the fiendish act with others of her sex, with little risk from the law or loss of social caste. It is, however, quite the reverse with the misguided woman, for, on disclosure of her frailty, there is immediate loss of social status, and her only alternative is either to seek personal or legal redress with the publication of her dishonor, or to remain quiescent and hide or endure her shame as best she can, or give herself up entirely to an abandoned life; and, when impregnated, to resort to abortion and commit child-murder in the wild effort to save her reputation, or submit to exposure and live the balance of her days under the ban of reproach as a degraded being—from which she may, perchance, be relieved by an acknowledged marriage, or, finally, as is so frequent, lapse into prostitution; while her innocent child is regarded as a waif, treated as an interloper in the human family, and branded with the ignominious titles of illegitimate, natural, bastard, etc.

The same injustice is also manifested in the general action of society, in the difference between the penalty inflicted in a simple breach of promise of marriage and seduction, for a man is apt to incur more social obloquy and greater legal punishment for the mere violation of a promise of marriage by word of mouth, than for the actual degradation of the woman by seductive cohabitation, the injury to the female being comparatively slight in the former to that of the latter—attended, as it always is, with loss of virtue, often, also, character, and, frequently, utter ruin; yet, the latter is treated so generally as the lesser, and the former the greater crime.

Now, this is obviously all wrong; yet, as a necessary result of the present imperfect social system, it is maintained as right, thus sanctioning evil and sustaining measures tending to the permanent degeneracy of the race, when by conforming more with the natural laws of life, such deterioration might readily be obviated. But to this end, as before intimated, there must be a radical change in the present views and customs of society respecting the natural affinities of the sexes, marriage, procreation, the rights of children, and the relations of life in general.

It is an acknowledged fact, that there are supreme natural laws governing the action of individuals and of the entire body politic, relating to the association of the sexes, births and deaths, as of everything else, and all issues in accordance therewith are necessarily legitimate, and cannot, without positive perversion, be regarded otherwise; hence, the usual conception of illegitimacy is, it appears to me, altogether wrong, being based upon false premises. Indeed, the prevailing ideas respecting this subject of illegitimacy are so erroneous as to be a disgrace to the vaunted intelligence, christianity, and civilization of the age. Thus, in the case of children, for instance, the very fact of their birth makes them legitimate in the natural order of things, the same as the production of the lower animals and other forms of life. But, even according to human estimate, there are very few children who could at all be considered otherwise than legitimate, and these only from the association of married men with single women or other men's wives, and single men with married women; all others are, unquestionably, entitled to immediate acknowledgement, and should be legitimatized by legal enactment recognizing the fact that *cohabitation and marriage go together*, the fact of sexual union being sufficient evidence of marital consent on the part of those thus indulging. This act of sexual conjugation being voluntary on the part of both male and female, and the essential element for procreation —the ultimate design of marriage—should be as conclusive of the marital union of men and women by the laws of man as they are by those of God; for, as generation occurs from sexual intercourse by the operation of the laws of life, without regard to human ordinances regulating the marital relation, it is, in reality, the only natural and true marriage physically considered, and, humanly, should be so acknowledged. Hence, as these so-called natural children are the fruit of a natural union of the sexes, and the product of natural marriage, by the operation of natural laws they have, *ipso facto*, the natural right to legitimacy, and should be legitimatized by human as they are by divine power, while their progenitors should be bound together by their marital tie, as well as obliged to recognize and provide for their offspring. Moreover, in those cases of forced conjunction, as in rape, the permanent association might also be made obligatory, if the injured female should decide that the union, thus involuntarily formed on her part, should be binding, although, even here, in the absence of such desire for its continuance, when impregnation occurred, justice would demand some provision for the legitimacy of the progeny, which might be effected by the application of the same rules as govern in divorce. Of course, if the decision of the woman be adverse to this coerced marriage, the ravisher should be severely punished, as usual. Furthermore, even in those apparently exceptional cases, arising from the intercourse of married men with single women or other men's wives, or single men with married women, the children should also be legitimatized by direct affiliation on the father.

These views are necessarily based upon the idea of monogamy —or the union of one man with one woman—and limited, of course, to those thus first cohabiting; or, secondarily, only as at present, by the loss of the former companion, all other connections being polygamous and illegal, to be punished as bigamy.

This plan is not, however, intended to interfere with the usual preliminary espousals, but designed rather to enlarge the sphere of legitimacy to its natural limits, in enforcing the recognition of an union contracted without preceding nuptial formalities, by legalizing marriages thus naturally consummated, and legitimatizing children engendered and born out of ordinary wedlock, thereby lessening seduction and diminishing illicit intercourse with its attendant evils.

The general adoption of this plan of legalizing marriages sexually consummated, would undoubtedly result in great good to all and harm to none, as, like the pledge by word of mouth, it would bind parties together only by their own act, force them to regard the sacred obligations thus incurred, and compel them to legitimatize and care for their progeny, the same as the more honorable always voluntarily do. To say nothing of the demoralization of men and women from illicit association, it is the height of cruelty to allow innocent beings to be thrust into existence tainted with dishonor, which, in the present state of public opinion, can never be effaced. Society has no right thus to brand the innocent with infamy and debar them from the normal relationship of life, whatever it may do with their offending parents. But, aside from the gross injustice of the act, and in view of the supremacy of the natural laws for the evolution of life, it is perfectly absurd to deny to children thus organized the claim to a legitimate birthright. It is just as foolish to thus proscribe these unfortunates, as it would be to reject the fruit from the tree because it had not grown in accordance with human ordinances, both being equally passive in their development within the great organism of life.

Thus, by the legal recognition of the fact that *the act of sexual conjugation constitutes marriage*, and binds the cohabiting parties as closely together in the marital relation as extraneous nuptial ceremonies now do, all disgrace would disappear, morality be promoted, weak women and innocent children protected, seduction, abortion, child-murder, prostitution, degradation, disease, and premature death diminished, and society be relieved of much of the evil which now so justly afflicts it, for permitting the present shameful state of things to exist.

It is, hence, both inexpedient and unwise, as well as unjust, to tolerate a system at variance with the principles of physiology, morality, religion, civilization, and infinite justice, which entails so much misery upon innocent beings and humanity in general, when, by the adoption of a more perfect jurisprudence, relief could be so readily obtained and mankind so greatly benefited.

Believing the preceding to be the true solution of a great social problem, and its practical application of inestimable value to humanity, it is the earnest hope of the writer that these few thoughts may enlist the sympathies and unite the efforts of all philanthropists to enact this suggestion into a rule of life, by proper education of society and suitable legislation on the subject, thus bringing human jurisprudence in entire harmony with the natural laws.

But as medical men are most familiar with the principles involved in this great question, and their influence is commensurate with their knowledge, they are especially invited to give this proposition their serious consideration, with a hearty coöperation, to insure the substantial realization of the proposed, or some better plan for thus benefiting their fellow-beings.

*Philadelphia, Nov. 2, 1867.*

